# A High Protein Calorie Restriction Diet Alters the Gut Microbiome in Obesity

**DOI:** 10.3390/nu12103221

**Published:** 2020-10-21

**Authors:** Tien S. Dong, Kayti Luu, Venu Lagishetty, Farzaneh Sedighian, Shih-Lung Woo, Benjamin W. Dreskin, William Katzka, Candace Chang, Yi Zhou, Nerea Arias-Jayo, Julianne Yang, Aaron Ahdoot, Zhaoping Li, Joseph R. Pisegna, Jonathan P. Jacobs

**Affiliations:** 1 The Vatche and Tamar Manoukian Division of Digestive Diseases, Department of Medicine, David Geffen School of Medicine at UCLA, Los Angeles, CA 90095, USA; tsdong@mednet.ucla.edu (T.S.D.); kaytiluu@gmail.com (K.L.); vlagishetty@mednet.ucla.edu (V.L.); farysedighian@ucla.edu (F.S.); wkatzka@gmail.com (W.K.); candacechang94@gmail.com (C.C.); zhouyisic@gmail.com (Y.Z.); narias@mednet.ucla.edu (N.A.-J.); jcyang1617@g.ucla.edu (J.Y.); aaronahdoot@ucla.edu (A.A.); jpisegna@mednet.ucla.edu (J.R.P.); 2 UCLA Microbiome Center, David Geffen School of Medicine at UCLA, Los Angeles, CA 90095, USA; zli@mednet.ucla.edu; 3 Division of Gastroenterology, Hepatology and Parenteral Nutrition, Veterans Administration Greater Los Angeles Healthcare System, Los Angeles, CA 90073, USA; benjamin.dreskin@va.gov; 4 Department of Medicine, Veterans Administration Greater Los Angeles Healthcare System, Los Angeles, CA 90073, USA; 5 Center for Human Nutrition, David Geffen School of Medicine at UCLA, Los Angeles, CA 90095, USA; shihlungwoo@gmail.com

**Keywords:** high protein diet, calorie restriction, microbiome, obesity, randomized controlled trial

## Abstract

Background: High protein calorie restriction diets have shown clinical efficacy for obesity, but the mechanisms are not fully known. The intestinal microbiome is a mediator of obesity and preclinical data support an effect of high protein diet (HPD) on the gut microbiome of obesity, but there are few studies in humans. Methods: To address this, we conducted a dietary intervention trial of 80 overweight and obese subjects who were randomized to a calorie-restricted high protein diet (HPD) (30% calorie intake) or calorie-restricted normal protein diet (NPD) (15%) for 8 weeks. Baseline dietary intake patterns were assessed by the Diet History Questionnaire III. Longitudinal fecal sampling was performed at baseline, week 1, week 2, week 4, week 6, and week 8, for a total of 365 samples. Intestinal microbiome composition was assessed by 16S rRNA gene sequencing. Results: At baseline, microbial composition was associated with fiber and protein intake. Subjects on the HPD showed a significant increase in microbial diversity as measured by the Shannon index compared to those on the NPD. The HPD was also associated with significant differences in microbial composition after treatment compared to the NPD. Both diets induced taxonomic shifts compared to baseline, including enrichment of *Akkermansia spp.* and *Bifidobacterium spp.* and depletion of *Prevotella spp.* Conclusion: These findings provide evidence that weight loss diets alter the gut microbiome in obesity and suggest differential effects of HPDs compared to NPDs which may influence the clinical response to HPD.

## 1. Introduction

Obesity has rapidly increased in prevalence in recent decades and now affects approximately one third of the population of the United States [1]. This epidemic has resulted in rising rates of type II diabetes mellitus, nonalcoholic fatty liver disease, dyslipidemia and arteriosclerosis as well as certain forms of cancer, including colorectal and pancreatic cancer. Dietary intervention and increased physical activity are the cornerstones of non-surgical management of obesity [2]. A wide range of diets have been studied to treat obesity, but a consensus has not been reached on an optimal macronutrient composition. A low-fat, high carbohydrate diet has traditionally been recommended; however this was challenged by alternative diets including those with increased protein and reduced carbohydrate intake. An early study reported that subjects randomized to ad libitum diets with 25% protein intake had greater weight and fat loss than subjects on a 12% protein diet over 6 months [3]. Further insight was provided by two large-scale randomized clinical trials—each with approximately 800 subjects—comparing the performance of isocaloric restriction diets with varying concentrations of carbohydrate, protein, and fat [4,5]. Subjects on diets with greater protein content had increased weight loss, reduced weight regain in the maintenance phase, and greater decrease in insulin. A meta- analysis of randomized studies comparing high protein and normal protein isocaloric restriction diets showed statistically significant decreases in weight, fat mass, and triglycerides with high protein diets (HPD) compared to normal protein diets (NPD) [6]. 

The underlying mechanisms of enhanced weight loss with an HPD are incompletely characterized. HPDs have been shown to promote satiety relative to isocaloric diets with more carbohydrates or fat [7,8]. This effect has been attributed to induction of satiety hormones including glucagon, glucagon-like peptide-1, and peptide YY 3-36 [8,9]. In clinical studies of ad libitum feeding, HPDs are associated with reduced energy intake relative to normal protein diets [3,10]. However, HPDs have also shown efficacy compared to isocaloric diets, indicating that other mechanisms are involved. It has also been proposed that weight loss is secondary to increased thermogenesis and preservation of lean body mass, maintaining resting energy expenditure [6,11,12]. This is supported by a study demonstrating that a high protein diet mitigates the reduction in resting and total energy expenditure that occurs on a calorie restricted diet [13]. 

There is now considerable evidence that the intestinal microbiome plays an important role in the pathogenesis of obesity. Germ-free mice have lower body fat and resistance to diet-induced obesity that are rapidly reversed upon microbial colonization [14,15]. Obese humans and mice have distinct intestinal microbiota from lean controls that induce increased body fat accumulation when transferred into germ-free mice compared to the microbiota of lean donors [16,17,18,19]. In addition, mice with microbiota alteration due to genetic defects in host immune pathways develop susceptibility to diet-induced obesity that can be transmitted to other mice by fecal transplantation [20,21]. Moreover, formerly obese mice have persistent changes in their microbiome that confer transmissible susceptibility to diet-induced obesity [22].

Long-term dietary patterns are correlated with microbiome composition and interventional diets can rapidly change microbial composition, raising the possibility that the microbiome is involved in the response to weight loss diets [23,24,25]. Higher dietary protein content in healthy volunteers has been associated with increased likelihood of a *Bacteroides spp*-predominant microbiota [24]. In animal models, HPDs have been shown to affect the microbiome of normal weight mice and rats after both short-term and prolonged feeding [26,27,28,29]. We recently demonstrated that switching rats with Western diet-induced obesity from a high fat to a high protein diet induces changes in the microbiome including enrichment of *Akkermansia muciniphila*, which had an inverse correlation with body fat mass [30]. This microbe has previously been reported to be enriched after bariatric surgery and to confer reduced adiposity and hyperglycemia in mice with diet-induced obesity [31,32,33]. 

We hypothesize that HPDs modulate the microbiome associated with human obesity, which may contribute to their clinical efficacy. Little data is currently available on the effects of high-protein weight loss diets on microbial composition in obese human populations. A non-randomized study of 19 obese participants placed on an HPD reported reduced *Roseburia*/*Eubacterium rectale* and *Bifidobacterium spp.* based on measurement of nine bacterial groups by fluorescence *in situ* hybridization [34]. In contrast, a more recent, randomized controlled study of two HPDs for 3 weeks with 12–13 overweight subjects per arm did not observe an effect of dietary intervention on the fecal microbiome using sequencing-based methodology [35]. To address this knowledge gap, we performed a randomized clinical study comparing the effect of isocaloric high and normal protein diets on the intestinal microbiome of a cohort of 80 overweight and obese subjects followed longitudinally for 8 weeks.

## 2. Materials and Methods 

### 2.1. Patient Recruitment

Patient recruitment occurred at the West Los Angeles VA Medical Center (ClinicalTrials.gov Identifier: NCT01146704). Patients were eligible for recruitment if they were between 20 and 75 years of age, BMI 27 to 40 kg/m^2^, non-smoker or stable smoking habits for at least 6 months prior to screening and agreement not to change such habits during the study. Patients were excluded if they had significant weight change of >3.0 kg in the month prior to screening, weight loss of >10 kg in the 6 months prior to screening, were on a calorie restriction diet (<1500 kcal/day) for a period of 4 months or more in the 12 months prior to screening, use of any other investigational drug(s) within 8 weeks prior to screening, abnormal baseline laboratory parameters (serum creatinine >1.6 mg/dL; alanine aminotransferase, aspartate transaminase, total bilirubin >2.0 times the upper limit of normal; triglycerides >500 mg/dL, total cholesterol >350 mg/dL, thyroid stimulating hormone outside of normal range), consumption of more than one alcoholic beverage per day, pregnancy or intention to become pregnant. All patients gave verbal and written consent to be part of the trial. The study was conducted in accordance with the Declaration of Helsinki, and the protocol was approved by the Greater Los Angeles VA Institutional Review Board (Project identification code: 2017-121121).

### 2.2. Enrollment and Randomization

Date of birth, sex, race, medical history, current medications, smoking and alcohol history were obtained during the screening visit. Metabolic syndrome was defined using the National Cholesterol Education Program (NCEP) Adult Treatment Panel III (ATP III) definition. Briefly, metabolic syndrome was defined as having any three of the following five criteria: waist circumference >40 inches in males or 35 inches in females, fasting glucose ≥100 mg/dL or history of diabetes mellitus on therapy, triglycerides ≥150 mg/dL or on therapy for hypertriglyceridemia, high density lipoprotein (HDL) <40 mg/mL in males or <50 mg/dL in females or was on therapy to raise their HDL, or blood pressure >130 mmHg systolic or >85 mmHg diastolic or was on therapy for hypertension [36]. After enrollment, patients were randomized to one of two diet groups using a random number generator. 

### 2.3. Dietary Intervention

Participants were randomized to an HPD (30% protein, 40% carbohydrate, 30% fat by calorie intake) or an NPD (15% protein, 55% carbohydrate, 30% fat) (i.e., control group). The diet was implemented in two phases: an initial macronutrient standardized diet without calorie restriction for 2 weeks then the same macronutrient standardized diet with a deficit of 500 calories from their calculated metabolic rate for 6 weeks. Basal metabolic rate was calculated using the InBody Scanner (Cerritos, CA, USA) body composition analysis, adjusting for routine daily activity by multiplying the basal metabolic rate by 1.2. Subjects received dietary counseling during their baseline visit on their assigned macronutrient standardized diet. After 2 weeks of a macronutrient standardized diet, both groups then received further dietary counseling on how to restrict their intake. Pea-based protein supplements (Nutrasumma, Phoenix, AZ, USA) were provided to the HPD group to achieve the target of 30% calories from protein in the HPD group. A control supplement containing dextrose that matched the calories of the protein supplement was given to the NPD group (Table 1). All patients, study coordinators, and medical doctors involved were blinded to the patient’s assignment.

### 2.4. Nutritional Assessments

Nutritional assessment was done at baseline using the Diet History Questionnaire III [37], a self-administered, semi-quantitative tool that queries the average frequency of consumption over the prior to one month of a list of food items. It is supported by the National Cancer Institute and was previously validated against other FFQs [38]. The extensive nutrient composition database that supports the questionnaire allows estimation of macronutrient intake. We used the United States Department of Agriculture’s (USDA) guideline for fiber and protein intake as the cutoffs for meeting recommended fiber and protein intake at baseline. For fiber, it meant taking in at least 38 g of fiber per day for males 50 years of age or younger and 25 g per day for females 50 years of age or younger. If the patients were over 50, the recommended daily fiber was 30 g and 21 g for males and females, respectively. For protein, we used the USDA’s recommended daily allowance of 0.8 g of protein per kilogram. 

### 2.5. Fecal Sampling

Participants were provided with kits for home sampling at baseline, week 1, week 2, week 4 (2 weeks of calorie restriction), week 6 (4 weeks of calorie restriction), and week 8 (6 weeks of calorie restriction). Stool samples were collected in a Para-Pak stool collection cup prefilled with 95% ethanol to fix the samples, allowing storage at room temperature for up to 2 weeks [39]. Samples were stored at −80 °C until undergoing DNA extraction using the ZymoBIOMICS DNA Microprep Kit (Zymo Research, Irvine, CA, USA) per the manufacturer’s protocol. Sequencing of the 253 base pair V4 region of 16S ribosomal DNA was performed as previously described using the Illumina NovaSeq 6000 to a depth of 250,000 reads per sample (primer set: 515f/806r) [40]. The sequences were processed with the DADA2 pipeline in R which assigns taxonomy using the SILVA 132 database [41]. After pre-processing in R, the data were imported into QIIME 2 version 2019.10 for further analysis [42]. Amplicon sequence variants were filtered if not present in at least 15% of all samples. Sequence depth ranged from 10,302 to 519,991 with a mean of 204,216 ± 71,888 per sample. 

### 2.6. Statistical Analysis

Baseline clinical characteristics were compared between the two arms using analysis of variance for continuous variables and chi-squared test for categorical variables. All means are presented along with their standard deviations. 

Microbiome data was analyzed for alpha diversity, beta diversity, and differential abundance of individual taxa. Because race was significantly associated with microbial outcomes at baseline, all analysis was adjusted for race. A composite category of race and ethnicity was used where we identified non-white Hispanics as a separate race category. Alpha diversity was calculated using the Shannon index (a metric of species evenness and richness) with data rarefied to 10,301 sequences. The significance of differences in alpha diversity was calculated by analysis of variance. Beta diversity was assessed using the DEICODE plugin in QIIME 2 which employs a robust Aitchison distance metric. This newer form of beta diversity metric accounts for the sparse compositional nature of microbiome data and has been shown to yield higher discriminatory power when compared to other used metrics such as UniFrac or Bray-Curtis [43]. Statistical significance of differences in beta- diversity was assessed using permutational multivariate analysis of variance (adonis package in R), with models including dietary intervention group, study time point, and subject. Baseline differences in taxa abundances were analyzed using DESeq2 in R (version 4.0.3, Vienna, Austria), which employs an empirical Bayesian approach to shrink dispersion and fit non-rarified count data to a negative binomial model [44]. For longitudinal samples, we used generalized linear mixed effects models implemented in the R package glmmTMB with the subject as a random effect [45]. *p*-values were adjusted for multiple hypothesis testing using the Benjamin–Hochberg procedure and the significance threshold was set at *p.adj* < 0.05.

## 3. Results

**Figure 1:**
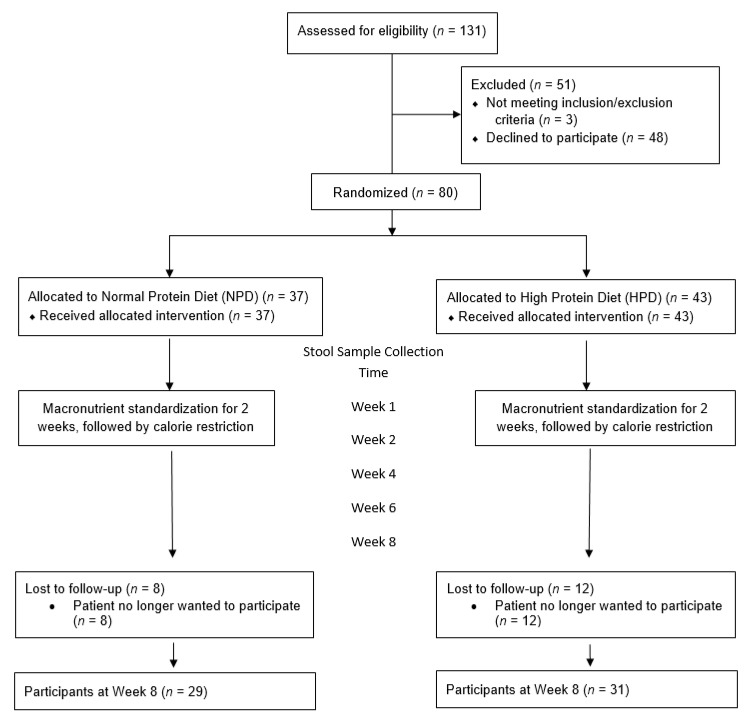
Study flow diagram.

A total of 131 patients were screened to be recruited in the study (Figure 1). Fifty-one patients were excluded. Three patients were excluded because they did not meet inclusion/exclusion criteria while the remaining 48 patients declined to participate. Eighty patients were enrolled and randomized into the two arms. At the end of the 8 weeks follow up period, there were 29 patients in the NPD arm and 31 patients in the HPD arm. The most common reason for drop out was that the patient no longer wanted to participate in the study due to time constraints.

The patients in the NPD and HPD groups did not differ by any baseline demographic or clinical parameter (Table 2). Because the study only included veterans receiving their medical care at the VA Greater Los Angeles Healthcare System, the majority of the patients (>75%) were male. The average age was 55.7 years in the NPD group and 55.9 years in the HPD group. The average body mass index was 34.6 in the NPD group and 34.9 in the HPD group. The two groups did not differ by the presence of diabetes mellitus, the presence of metabolic syndrome or race. Baseline macronutrient and fiber intake was also not statistically different between the two groups. At the end of 8 weeks, the HPD lost more weight on average than the NPD group (3.46 kg vs. 2.83 kg), though the results were not statistically significant (*p*-value = 0.34). 

**Figure 2:**
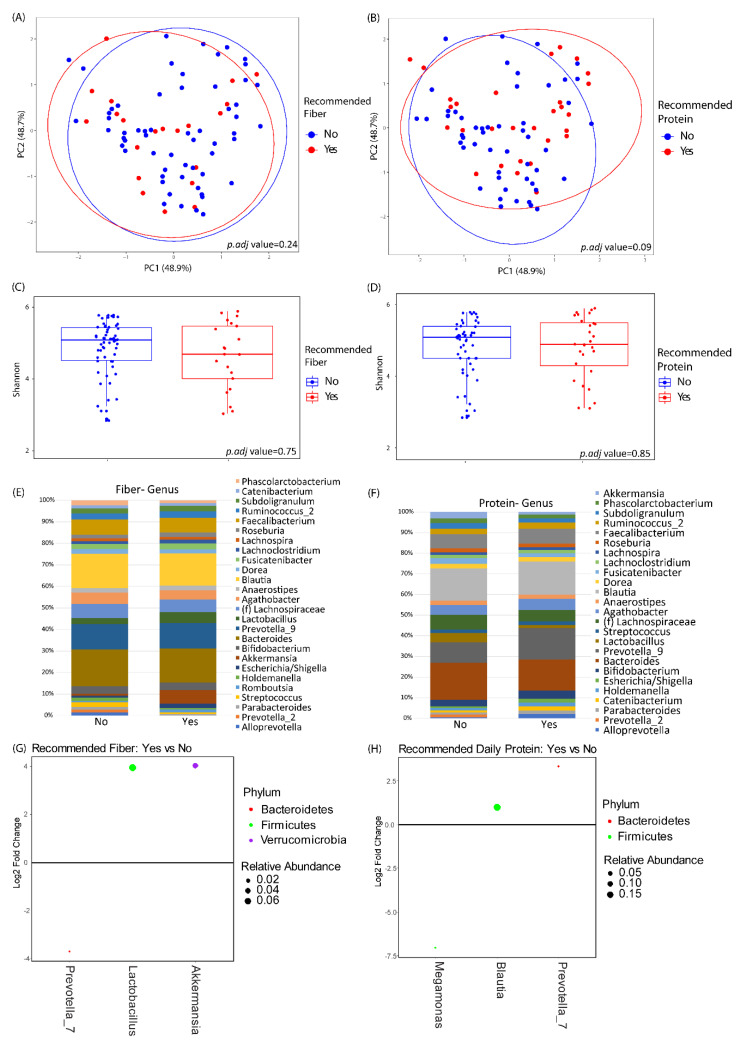
Baseline microbiome differences by protein and fiber intake. (**A**,**B**) Principal coordinates analysis plots of microbial community makeup by (**A**) recommended fiber intake and (**B**) recommended protein intake. (**C**,**D**) Shannon index of microbial diversity by (**C**) recommended fiber intake and (**D**) recommended protein intake. (**E**,**F**) Taxonomic summary plots by (**E**) recommended fiber intake and (**F**) recommended protein intake. Only genera with ≥1% relative abundance are shown. (**G**,**H**) Differentially abundant genera by DESeq2 analysis between subjects at baseline stratified by (**G**) recommended fiber intake or (**H**) recommended protein intake. Genera that are above 0 represent genera that have a higher abundance in patients who are taking in the recommended amount of fiber or protein as compared to those that are not. Dots are colored by phylum and sized by mean relative abundance across all samples.

Baseline microbiome profiles did not differ between the two groups by either alpha or beta diversity analysis (data not shown). There were also no significant differences of baseline microbiome diversity or composition in relationship to baseline fiber or protein intake (Figure 2). Baseline fiber and protein intake was classified as either meeting the recommended daily intake according to USDA guidelines or not meeting the recommended daily intake. The baseline abundances of genera with greater than 1% mean relative abundance are shown in Figure 2E for subjects in the study, stratified by baseline fiber and protein intake. Differential abundance testing identified three genera that significantly differed between those meeting the daily recommended intake of fiber versus those that did not. *Akkermansia spp.* and *Lactobacillus spp.* were enriched in patients with higher fiber intake, and *Prevotella_7 spp.* was depleted in patients with higher fiber intake. Similarly, three genera were found to be differentially abundant between those meeting the daily recommended intake of protein versus those that did not. *Prevotella_7 spp.* and *Blautia spp.* were elevated in patients with higher protein intake, and *Megamonas spp.* was lower in patients with higher protein intake. 

**Figure 3:**
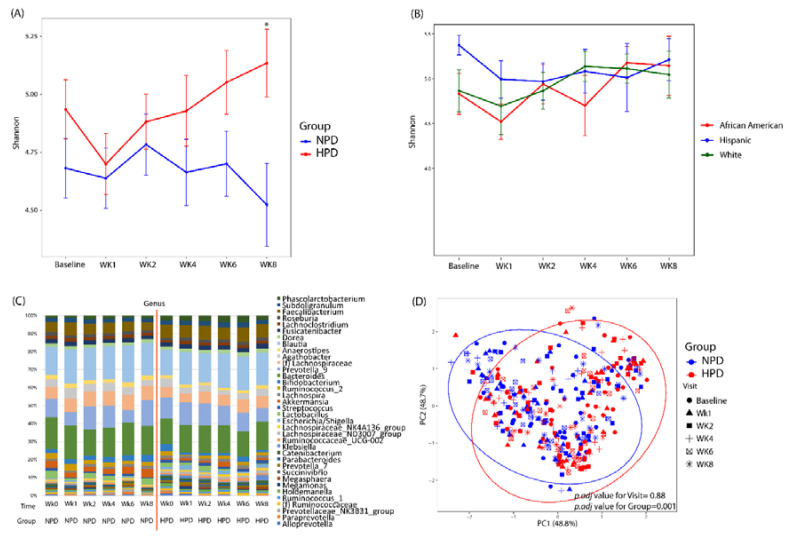
High protein and calorie restriction effects on microbial diversity and composition. (**A**) Shannon index across study visits and by intervention group. (**B**) Shannon index for the HPD group divided by race/ethnicity. (**C**) Taxonomic summary plots of genera by study visit and by group. Only genera with a relative abundance ≥1% are shown. (**D**) Principal coordinates analysis of microbial commnuity composition colored by group with shapes representing study visit. NPD: Normal protein diet. HPD: High protein diet. Wk: Week. * Denotes *p*-value < 0.05 between the two arms.

After patients were randomized and placed on either an HPD or NPD diet with calorie restriction, significant changes in microbial diversity and composition were observed (Figure 3). There was an initial decrease in alpha diversity as assessed by the Shannon index when patients transitioned to a standardized macronutrient diet (Figure 3A). This was evident in both the NPD and HPD group. However, at the end of 8 weeks (6 weeks of calorie restriction), the alpha diversity of the NPD group remained unchanged compared to baseline while the HPD group significantly increased. At the end of the 8-week trial, patients on the HPD had a significantly higher Shannon index as compared to those on the NPD (*p*-value < 0.05). This increase in diversity was apparent in the white and African-American subjects in the HPD group but not the Hispanic subjects (Figure 3B). The taxonomic summary of the two groups across the various time points is shown in Figure 3C. Beta diversity analysis demonstrated that there was a statistically significant difference in microbial composition between the HPD and NPD groups when considering all study time points together and adjusting for subject effects (*p.adj* value = 0.001) (Figure 3D). There was no beta diversity difference by study visit (*p.adj* value = 0.88).

**Figure 4:**
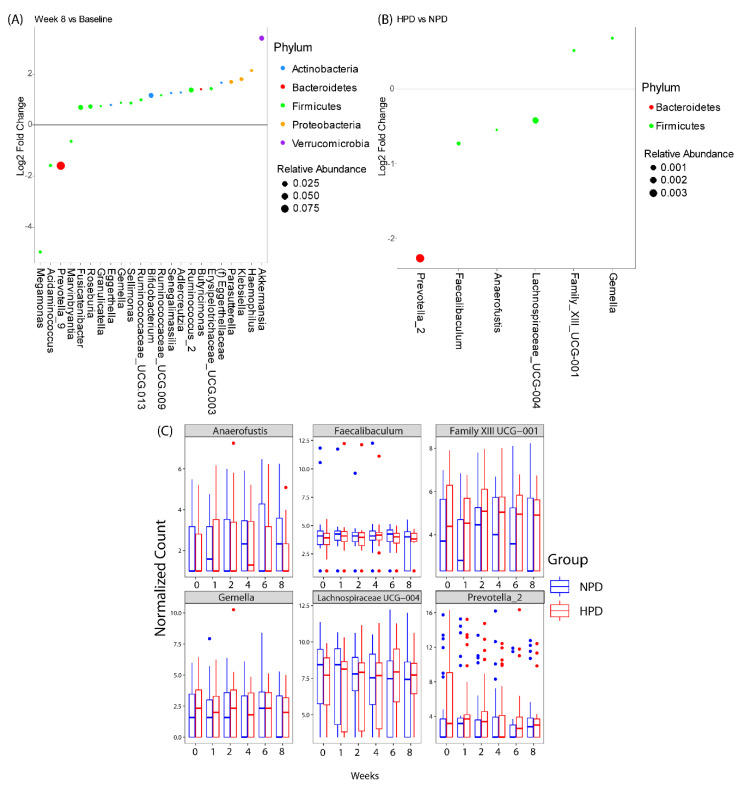
Differentially abundant taxa with the calorie restriction diets. Genera that were significantly different (**A**) at the end of week 8 as compared to baseline independent of study arm or (**B**) between high protein diet (HPD) and normal protein diet (NPD) group independent of study visit (incorporates data from all time points). Genera that are above 0 represent genera that have a higher abundance in patients at week 8 or on a HPD as compared to baseline or NPD, respectively. Dots are colored by phylum and sized by mean relative abundance across all samples. (**C**) Box plots showing DESeq2 normalized counts of the 6 genera that were differentially abundant between HPD and NPD across time points.

Differential abundance testing demonstrated that there were genera that changed over the course of the study irrespective of diet, and genera that changed specifically due to higher protein intake (Figure 4). Twenty-three genera were either enriched or depleted at 8-weeks as compared to baseline, independent of protein intake. The majority of the genera belonged to the phylum Firmicutes. The three genera with the highest relative abundance were *Akkermansia*, *Bifidobacterium*, and *Prevotella_9*. *Akkermansia spp.* and *Bifidobacterium spp.* were elevated at 8-weeks as compared to baseline while *Prevotella_9* was decreased at 8-weeks as compared to baseline. The HPD intervention resulted in differential abundance of six genera as compared to the NPD group when considering all study time points together. The majority of the genera belonged to the phylum Firmicutes. The three genera with the highest relative abundances were *Prevotella_2*, *Faecalibaculum*, *Lachnospiraceae_UCG- 004*. All three genera were underrepresented in patients on the HPD as compared to the NPD group. The genus that was most strongly enriched in the HPD group was *Gemella*.

## 4. Discussion

We demonstrated that isocaloric calorie restriction diets significantly impact the gut microbiome of overweight and obese participants in a manner that depends on dietary protein intake. The HPD notably induced an increase in intestinal microbial diversity relative to the NPD that was primarily observed in the white and African-American subgroups. This difference between Hispanics and non- Hispanic whites and African-American subgroups could be due to fact that the Hispanic subgroup started with a higher microbial diversity than the other ethnic groups. Since this group of Hispanic volunteers had higher microbial diversity, it is possible that an HPD was unable to increase it further. Studies have shown that Hispanics who have immigrated to the US at a later age have higher diversity than those who were either born in the US or who have immigrated when they were children [46]. While we did not ask about immigration history in our initial assessment, it is possible that a few patients in our Hispanic subgroup may have been relatively recent immigrants to the US or were living with members who have recently immigrated, thus causing them to have higher microbial diversity at baseline. Microbial diversity in the intestine is associated with health, and loss of diversity occurs with many disease states such as inflammatory bowel disease [47]. A meta- analysis of microbiome studies of obesity found a significant decrease in microbial diversity in obese individuals by measures including the Shannon index [48]. Increased diversity on an HPD may be a marker of restoration of a metabolically more favorable microbial state. Obesity is not only associated with low microbial diversity at the taxonomic level but also with low bacterial gene richness, which has been linked to inflammation and metabolic dysfunction [49]. Interestingly, an HPD was reported in a non-controlled study to increase microbial gene richness in obese subjects, suggesting that the increased compositional diversity that we observed with an HPD could also correspond to greater microbial gene richness [50]. 

The HPD also induced a significant shift in overall microbial composition which differed from that observed in the NPD control. At the level of individual taxa, this corresponded to a reduction in *Prevotella_2 spp.*, a less-abundant genus within the Prevotellaceae family compared to the *Prevotella_9* genus which is dominant within the gut microbiota of Western populations [51]. *Prevotella_2 spp.* has previously been reported to be associated with cardiovascular disease risk, suggesting that it has adverse metabolic effects and that its depletion by an HPD could have beneficial effects [52]. The HPD also enriched for *Gemella spp.*, a group of Gram positive cocci that is a significant constituent of the microbiome of the upper gastrointestinal tract but is present at much lower levels in the colon [53]. Its enrichment in the fecal microbiota of subjects receiving an HPD suggests that this dietary change is able to modify the typical colonization range of *Gemella spp.* to allow extension into distal regions of the digestive tract. It is unclear if there is any functional consequence of this taxonomic shift as there is no literature on the role of intestinal *Gemella spp.* in obesity. Little is known about the human members of the remaining four genera that were differentially abundant on an HPD.

The mechanism by which an HPD alters the microbiome and increases richness may involve increased delivery of dietary amino acids to the distal gastrointestinal tract for bacterial fermentation. While intestinal microbes under most conditions largely derive nitrogen from endogenous sources (e.g., host glycoproteins), high dietary intake can shift the balance such that dietary protein becomes the dominant nitrogen source, thereby favoring some bacteria over others [54]. In support of this model of increased bacterial utilization of dietary protein on an HPD, a study of overweight and obese subjects on an HPD observed increased fecal levels of metabolites such as branched chain fatty acids, p-cresol, and 4-hydroxyphenylpyruvic that are consistent with amino acid fermentation [55].

It is unknown whether the effects of an HPD on the microbiome of obesity are primarily dependent on the proportion of caloric intake comprised of protein or the source of dietary protein. In our study, we prescribed a combination of protein from lean meats and plant sources, including a protein supplement derived from peas, to provide a diversity of protein sources. It has previously been reported that an animal-based vs. plant-based diet can differ greatly in their effects on the microbiome, but this may have arisen due to differences in nutritional content such as fiber rather than the source of dietary protein [25]. Existing data from preclinical protein supplementation studies suggest that distinct types of protein can have differing effects on the gut microbiome [56]. For instance, hamsters supplemented with soy protein had greater microbial diversity and altered composition including increased *Bifidobacteria spp.* compared to hamsters supplemented with milk protein [57]. 

While the microbiome effects of the HPD significantly differed from that of an NPD, both diets had a shared signature of taxonomic shifts with particularly striking induction of *Akkermansia spp*. This is unlikely to be an effect of calorie restriction itself, as studies of calorie restriction diets have not found evidence of changes in microbial diversity or composition [58,59]. We found that baseline levels of *Akkermansia spp.* were associated with fiber intake, suggesting that dietary changes shared between both diets including the emphasis on deriving dietary carbohydrate intake from high-fiber sources may have driven this change. Increased *Akkermansia spp.* is anticipated to have beneficial metabolic effects on adiposity and insulin resistance based on preclinical and human studies [31,32,33]. Both diets also increased fecal levels of *Bifidobacterium spp*. This genus has been reported to reduce insulin resistance and inflammation in animal models of metabolic syndrome [60,61]. Interestingly, individuals with a lactase genotype associated with increased *Bifidobacterium spp.* abundance had greater adiposity reduction on an HPD than those without this variant, suggesting that *Bifidobacterium spp.* is an important therapeutic target of HPDs [62]. In addition, both diets suppressed the predominant *Prevotella* genus, *Prevotella_9*. *Prevotella spp.* abundance has been reported to be enriched in obesity in a large cohort of Hispanics living in the United States and has also been associated with diabetes as well as progression of fatty liver disease [46,63,64]. Its suppression by both HPD and NPD may contribute significantly to the beneficial clinical outcomes with these weight loss interventions.

Our study has several key advantages compared to prior studies. It included a larger number of subjects, greater racial/ethnic diversity, frequent fecal sampling during longitudinal follow-up (six samples over 8 weeks), and high-depth sequencing with NovaSeq to allow for detection of rare taxa. However, it does have several limitations. The use of dietary counseling rather than meal replacement introduces additional variation into the study due to incomplete adherence and heterogeneity of patient dietary choices. The cohort was male-predominant, so the results may not be applicable to females. In addition, while the study was larger than prior ones, it did not have enough subjects to be powered to detect a difference in clinical outcomes such as weight loss. Finally, the microbiome analysis was based on 16S rRNA sequencing, which provides data on microbial composition but not on microbial gene content or function.

In summary, we provide evidence that HPDs modulate intestinal microbiome composition in obesity. These findings support the hypothesis that microbial changes influence the outcomes of high protein dietary interventions. Additional studies with larger cohort size and longer duration are required to determine to what extent longitudinal changes in the microbiome are associated with clinical outcomes on an HPD. Incorporation of metagenomics and metabolomics to provide functional microbiome data may provide insight into mechanistic pathways that could undergo further study in preclinical models. Additional future directions could include a comparison of different protein sources and/or HPDs of varying protein content. Further understanding of the link between the microbiome and the beneficial effects of a high protein diet may spur development of therapies for obesity that directly target the microbiome to complement dietary modification.

## Figures and Tables

**Table 1:** Meal plan between the study arms and supplement characteristics per serving.

	NPD	HPD
Meal Plans (% of Total Calories/Day) Including Supplement		
Grains/Starch	20	10
Vegetables	15	15
Protein	15	30
Fruit	20	15
Fats and Oils	30	30
Supplement Characteristics per Serving		
Calories	100	100
Total Fat	0 g	1 g
Total Carbohydrate	22 g	1 g
Protein	0 g	20 g
Sodium	160 mg	290 mg
Potassium	45 mg	35 mg

NPD: Normal Protein Diet; HPD: High Proteing Diet.

**Table 2:** Baseline cohort characteristics.

	NPD (*n* = 37)	HPD (*n* = 43)	*p*-Value
Gender (% Males)	78.4	76.7	0.86
Age (years) (SD)	55.7 (11.4)	55.9 (10.1)	0.91
BMI	34.6 (5.1)	34.9 (4.5)	0.8
Weight (kg) (SD)	104.3 (18.1)	104.7 (18.4)	0.93
Presence of DM (%)	27.0	32.6	0.59
Presence of Metabolic Syndrome (%)	32.4	41.9	0.39
Race (%)			
White (*n* = 34)	56.8	30.2	0.15
African American (*n* = 26)	27.0	37.2	
Hispanic (*n* = 17)	13.5	27.9	
Asian (*n* = 2)	2.7	2.3	
Other (*n* = 1)	0.0	2.3	
Average Weight Change at Week 8 (kg) (SD)	−2.83 (2.25)	−3.46 (2.67)	0.34
Baseline Diet			
Total Calories (kcal) (SD)	2561.1 (1580.7)	2003.7 (1629.9)	0.13
Protein (% of total kcal/day) (SD)	16.4 (3.9)	17.7 (4.3)	0.16
Fat (% of total kcal/day) (SD)	37.3 (6.7)	37.5 (6.9)	0.93
Carbohydrate (% of total kcal/day) (SD)	47.4 (7.5)	45.8 (8.9)	0.39
Fiber (g) (SD)	24.7 (14.7)	19.2 (17.2)	0.14

HPD: High protein diet. NPD: Normal protein diet. DM: Diabetes. BMI: Body Mass Index. SD: Standard deviation. Kcal: kilocalories, g: grams.
